# Effects of cassava waste- and tofu waste-based concentrate formulations on feed intake, nutrient digestibility, growth performance, and economic returns of Madura and Madrasin bulls under smallholder production systems

**DOI:** 10.14202/vetworld.2026.2787-2801

**Published:** 2026-07-10

**Authors:** Kusmartono Kusmartono, Mashudi Mashudi, Poespitasari Hazanah Ndaru, Aprilia Dwi Kartika2 Karen Jean Harper, Stephen Todd Morris

**Affiliations:** 1 Animal Science Study Program, Department of Animal Science, Faculty of Animal Science, Universitas Brawijaya, Malang 65145, Indonesia.; 2 Doctoral Program of Animal Science, Faculty of Animal Science, Universitas Brawijaya, Malang 65145, Indonesia.; 3 School of Health, Medical and Applied Sciences, Central Queensland University, Rockhampton, QLD 4702, Australia.; 4 School of Agriculture and Environment, College of Science, Massey University, Palmerston North New Zealand.

**Keywords:** agricultural by-products, beef cattle, cassava waste, feed formulation, income over feed cost, Madrasin bulls, Madura bulls, smallholder production systems

## Abstract

**Background and Aim:**

Feed scarcity and the high cost of commercial concentrates remain major constraints to beef production under Indonesian smallholder systems. Agricultural by-products such as cassava and tofu waste offer potential low-cost alternatives, but information on their combined utilization and breed-specific responses remains limited. This study aimed to evaluate the effects of different cassava waste- and tofu waste-based concentrate formulations on feed intake, nutrient digestibility, growth performance, and economic returns in indigenous Madura and Madrasin (Madura × Limousin) bulls.

**Materials and Methods:**

Forty bulls (20 Madura and 20 Madrasin; 2.0–2.5 years old) were used in a 2 × 4 factorial experiment arranged in a randomized complete block design. Four concentrate formulations containing increasing levels of cassava waste (19%-34%) and decreasing levels of tofu waste (20%-5%) were supplied together with native grass. Feed chemical composition was determined, and dry matter intake (DMI), nutrient intake, digestibility coefficients, liveweight gain (LWG), feed conversion ratio (FCR), and income over feed cost (IOFC) were measured. Data were analyzed using mixed-model analysis of variance.

**Results:**

Increasing cassava waste inclusion enhanced DMI, with the highest value observed in Madrasin bulls receiving the T4 diet (7.19 kg/day). Dry matter digestibility ranged from 75.06% to 79.77%, whereas organic matter digestibility ranged from 76.77% to 81.21%. Crude protein digestibility remained relatively stable among treatments. Despite differences in intake and digestibility, LWG (0.62-0.83 kg/day) and FCR (7.58-9.64) were not significantly affected by dietary treatments. Madrasin bulls consistently exhibited greater nutrient intake, superior fiber utilization, higher average daily gain, and greater economic returns than Madura bulls. IOFC reached a maximum of IDR 44,456/day in Madrasin bulls. Importantly, increasing cassava waste from 19% to 34% did not adversely affect growth performance or profitability, indicating that dietary flexibility could be achieved without compromising productive efficiency.

**Conclusion:**

Cassava waste and tofu waste can be effectively combined in concentrate formulations for beef cattle under smallholder conditions. Inclusion of cassava waste up to 34% maintained growth performance, feed efficiency, and profitability while reducing dependence on commercial concentrates. Madrasin bulls exhibited superior productive and economic performance; however, balanced breeding strategies are required to enhance productivity while supporting conservation of the indigenous Madura cattle. These findings provide practical evidence for developing resilient, low-cost, and sustainable feeding systems based on locally available agricultural by-products.

## INTRODUCTION

Indonesia’s smallholder cattle production system is a critical pillar of national food security, rural livelihoods, and the preservation of genetic resources. Smallholders contribute approximately 90% of domestic cattle production, yet national beef output meets only about 45% of total demand, creating a persistent supply deficit that increases reliance on imports [1, 2]. This structural imbalance imposes considerable pressure on rural producers, who typically operate on limited land and capital and whose productivity is constrained by feed scarcity, fluctuating input costs, and restricted market access [3]. The dual challenge of increasing production while maintaining livelihood stability underscores the importance of strengthening smallholder feeding systems.

A central limitation in Indonesian smallholder cattle systems is the inadequate year-round access to high-quality feed. Feed shortages are intensified by competition for land, seasonal variability, and the high cost of commercial concentrates, which many smallholders cannot afford [2]. Consequently, producers rely heavily on low-quality natural pastures and crop residues that provide insufficient nutrients to sustain optimal growth, particularly in intensively fattened cattle. This nutritional gap suppresses average live weight gain (LWG), compromises reproductive performance, and adversely affects animal health. Studies have demonstrated that introducing improved feed resources, such as forage tree legumes and high-quality silage, yields substantial gains in smallholder systems. For example, the use of *Leucaena* has been reported to double cattle growth rates under traditional management [4, 5]. Despite their potential, the adoption of such innovations remains uneven because of knowledge gaps and limited local processing capacity.

Indigenous cattle breeds play an essential role in this context, contributing not only to household income but also to the ecological and cultural resilience of livestock-based livelihoods. Breeds such as Madura cattle are deeply integrated into local production environments and are valued for their adaptability to harsh tropical conditions and tolerance to parasitic challenges. These animals can maintain productivity under limited feed availability [6, 7], making them indispensable to smallholder production systems. Madura cattle are small, hardy, and heat-tolerant, typically reaching market weights of 200–300 kg, whereas Madrasin (Madura × Limousin) crossbreds are larger, faster growing, and more feed-efficient, often achieving market weights of 350–450 kg. Although Madura cattle excel in adaptation to tropical environments, Madrasin cattle have been selected primarily for enhanced productivity [8]. Consequently, safeguarding indigenous genetic resources has become increasingly important because uncontrolled crossbreeding threatens population integrity and long-term breed sustainability.

Another important challenge involves the strategic utilization of agricultural by-products as alternative feed resources. Across Southeast Asia, farmers routinely incorporate by-products derived from rice, palm oil, fruit, and tuber processing into ruminant diets, thereby providing inexpensive sources of fermentable energy and structural fiber [3, 9]. In Indonesia, particularly in East Java, abundant agro-industrial residues, including cassava waste, tofu waste, rice bran, and copra meal, remain underutilized due to limited information on their nutritional value, processing requirements, and optimal inclusion rates. Improving the utilization efficiency of these resources is essential for reducing feed costs while maintaining adequate nutrient supply. Previous studies have shown that appropriate processing and ration formulation can markedly improve the nutritive value of agricultural by-products and stabilize feed availability throughout the year [9–11].

The incorporation of local by-products into smallholder feeding systems aligns with Indonesian national strategies to reduce dependence on commercial concentrates and imported feed ingredients. Nevertheless, the successful adoption of by-product-based feeding systems requires a comprehensive evaluation of their nutritional characteristics, effects on feed intake and digestibility, and economic feasibility. Cassava waste, particularly in the form of ground tubers, provides abundant fermentable energy but contains limited crude protein (CP) and may contain anti-nutritional compounds such as cyanogenic glycosides. In contrast, tofu waste is rich in protein and lipids but may contain anti-nutritional substances, including trypsin inhibitors, phytates, and phytoestrogens, thereby necessitating careful ingredient balancing to maintain ruminal function and microbial protein synthesis. No previous study has simultaneously manipulated the proportions of cassava waste and dried tofu waste in a single concentrate formulation while comparing responses across specific cattle breeds. Therefore, understanding the interactions among these ingredients in rations is essential for optimizing nutrient utilization, ruminal fermentation, and growth performance.

Breed-specific responses to by-product-based diets represent another important aspect. Crossbred cattle, such as Limousin-influenced Madrasin bulls, generally exhibit superior growth performance compared with indigenous breeds due to greater rumen capacity, enhanced microbial activity, and improved energy-partitioning efficiency [12–14]. These physiological advantages are often associated with greater feed intake, improved fiber digestibility, and higher average daily gain under supplemented feeding systems. Although crossbreds provide greater short-term economic returns, their higher nutrient requirements and lower adaptability to resource-constrained environments may limit their suitability for some smallholders and accelerate the erosion of indigenous Madura cattle genetic resources. Empirical information comparing the productive and economic performance of crossbred and indigenous cattle fed locally available feed resources under smallholder conditions remains scarce. Consequently, comparative evaluations are needed to quantify breed differences under standardized feeding regimes based on local by-products.

Despite increasing interest in the use of agricultural by-products in tropical livestock production, several important knowledge gaps remain. First, direct comparisons between indigenous Madura cattle and Madrasin crossbreds receiving locally formulated rations are limited, thereby restricting evidence-based decision-making for farmers and breeding programs. Second, previous studies have generally investigated cassava waste and tofu waste separately, leaving uncertainty regarding their combined effects on feed intake, nutrient digestibility, growth performance, and profitability. Third, most studies have focused on either nutritional characteristics or productive responses alone and have rarely integrated feed characterization, controlled feeding experiments, and economic evaluation within a single framework. Moreover, information generated under practical smallholder production environments remains limited. These shortcomings hinder the development of scientifically validated feeding strategies that can improve productivity while supporting the conservation of indigenous cattle genetic resources.

Addressing these limitations is essential for improving the productivity and sustainability of Indonesian smallholder cattle systems. Therefore, the present study was conducted to characterize the nutrient composition of locally available feed ingredients and to evaluate the effects of different inclusion levels of cassava and tofu waste on feed intake, nutrient digestibility, growth performance, and economic returns in Madura and Madrasin bulls. Bangkalan Regency was selected as the study area because it represents the traditional center of Madura cattle production and provides abundant access to agricultural by-products, although optimized feeding recommendations remain limited. By integrating detailed feed characterization, *in vivo* animal responses, and economic analysis based on income over feed cost (IOFC) within a 2 × 4 factorial design under communal smallholder conditions, this study provides novel and location-specific evidence for developing flexible, low-cost, and sustainable feeding strategies that simultaneously enhance productivity and support indigenous breed conservation.

It was hypothesized that optimizing the ratio of cassava to tofu waste in a standardized concentrate would improve nutrient intake, digestibility, and growth performance. Furthermore, it was anticipated that Madrasin bulls would exhibit superior growth performance because of their physiological advantages, whereas indigenous Madura cattle would maintain competitive economic efficiency based on IOFC when fed locally available agricultural by-products. Ultimately, this study contributes to broader efforts to strengthen feed self-sufficiency, improve cattle productivity, and safeguard indigenous cattle resources in Indonesia’s evolving livestock sector. The findings are expected to support policy development, extension programs, and on-farm decision-making, thereby helping smallholders overcome feed shortages, increasing input costs, and crossbreeding pressures while promoting more sustainable, profitable, and resilient production systems.

## MATERIALS AND METHODS

### Ethical approval

All procedures involving animals were reviewed and approved by the Animal Ethics Committee, Faculty of Veterinary Medicine, Universitas Brawijaya, Malang, Indonesia (Approval No. 57-KEP-FKHUB-2025). The study was conducted in accordance with national guidelines governing the care and use of animals in research and complied with institutional standard operating procedures for handling bulls. Throughout the experimental period, all animals were maintained under appropriate housing, feeding, and veterinary management to ensure their welfare. The study also complied with Indonesian regulations concerning animal welfare and internationally accepted principles for the ethical use of animals in scientific research.

### Study period and location

The study was conducted from January to December 2025 and comprised both the adaptation and experimental periods, following the procedures described by Moran [15]. The study was conducted in Bangkalan Regency, East Java Province, Indonesia (7°01′51″S, 112°44′42″E), at an altitude of approximately 56 m above sea level. This region is characterized by smallholder-based cattle production systems in which forage availability is often limited and locally available agricultural by-products are widely utilized, making it an appropriate location for evaluating locally formulated concentrate rations. During the 21-day adaptation period, bulls were gradually acclimatized to the concentrate mixtures and communal housing facilities. Maize stover was offered during the first 3 days, followed by a gradual increase in concentrate allowance during the subsequent 18 days to facilitate ruminal adaptation [16]. The experimental period lasted for 90 days after completion of the adaptation phase. All animal-related procedures were performed in smallholder-managed communal stalls modified to permit individual feeding, thereby ensuring experimental accuracy without disrupting the traditional production environment. Nutrient analyses were conducted at the Nutrition Laboratory, Faculty of Animal Science, Universitas Brawijaya, Malang, Indonesia, under standardized laboratory conditions.

### Experimental animals

Forty bulls, consisting of 20 Madura and 20 Madrasin animals aged 2.0–2.5 years, were used in the study. The breed-specific initial live weight (LW; mean ± standard deviation) was 288 ± 18.4 kg for Madura bulls and 322 ± 22.1 kg for Madrasin bulls, with an overall mean LW of 305 ± 23.6 kg. Before the experiment, all animals underwent clinical examination by a veterinarian and were confirmed to be healthy. No vaccinations were administered because no endemic disease outbreaks were reported during the study period. Animals were purchased from regional cattle markets in Bangkalan with documented farmer origin and health records to ensure traceability. Each bull was fitted with a neck collar tag for identification throughout the experiment.

Prior to the adaptation and feeding phases, internal parasites were controlled with Nitroxinil (Fluconix® 340; Interchemie Werken "De Adelaar" B.V., Castenray, the Netherlands) at a dose of 1.5 mL/50 kg LW. Subsequent mentions should refer to Fluconix® 340 or Interchemie Werken "De Adelaar" B.V. only. LW measurements were performed every 2 weeks in the morning before feeding to facilitate adjustment of feed allowances according to current LW.

### Stall design and management conditions

Animals were housed in communal cattle stalls equipped with separators to enable individual feed intake measurements. Separate feed troughs were provided for forage and concentrate. The pens were roofed and had concrete flooring to minimize thermal and environmental stress. Fresh drinking water was provided *ad libitum* throughout the study.

### Study design and feeding regimes

The experiment was conducted using a randomized complete block design with a 2 × 4 factorial arrangement, comprising two breeds (Madura and Madrasin) and four concentrate treatments. A total of 40 bulls (20 Madura and 20 Madrasin) were allocated to the treatments [17]. Initial LW served as the blocking factor, with five blocks established according to LW classes ranging from 260 to 350 kg. Animals were randomly assigned to one of four dietary treatments, resulting in five animals per breed × treatment combination. Thus, the block term in the statistical model represented the initial LW class. Individual animals were considered the experimental units because they were managed and fed individually and feed intake and refusals were measured separately. This experimental design was based on previous studies [18, 19].

All bulls received native grass at 0.5% of LW as the basal diet and one of four concentrate mixtures at 2.25% of LW on a DM basis. Native grass was harvested at the vegetative stage, chopped into 3–5 cm lengths, and offered fresh. The dietary treatments were as follows:

1. T1 = native grass (0.5% LW) + C1 (2.25% LW)

2. T2 = native grass (0.5% LW) + C2 (2.25% LW)

3. T3 = native grass (0.5% LW) + C3 (2.25% LW)

4. T4 = native grass (0.5% LW) + C4 (2.25% LW)

The ingredient composition of the concentrate mixtures is presented in Table 1. The principal difference among treatments was the progressive increase in cassava waste from 19% in C1 to 34% in C4, accompanied by a corresponding reduction in tofu waste from 20% to 5%. Rice bran, copra meal, broken corn, and mineral mixture remained constant among treatments. Concentrates were mixed daily using a mechanical mixer, stored in sealed plastic containers at room temperature, and offered twice daily at 08:00 and 15:00 h. Unlike many station-based studies that use pelleted or ensiled feeds, the present study employed fresh local by-products at inclusion levels representative of farmers' practices while maintaining near-isoenergetic concentrate formulations.

**Table 1:** Ingredient composition of the concentrate mixtures (% DM basis).

**Ingredients**	**C1**	**C2**	**C3**	**C4**
Rice bran	20	20	20	20
Cassava waste	19	24	29	34
Tofu waste	20	15	10	5
Copra meal	30	30	30	30
Broken corn	10	10	10	10
Mineral mix	1	1	1	1
Total	100	100	100	100

### Feed ingredients and chemical composition analyses

The ingredients used in this study included native grass, rice bran, cassava waste, tofu waste, copra meal, broken corn, and a mineral mix. Samples of each ingredient were analyzed for dry matter (DM), organic matter (OM), crude protein (CP), ether extract (EE), and crude fiber (CF) according to the methods described by AOAC [20]. Total digestible nutrient (TDN) values were calculated according to the equation proposed by Moran [16]. Acid detergent fiber (ADF) and neutral detergent fiber (NDF) contents were determined following the procedure described by Van Soest *et al.* [21].

### Feeding procedures and intake measurements

Feed allowances were adjusted weekly according to changes in LW to maintain the desired feeding rates. Forage and concentrate were offered separately to minimize selective feeding. Feed refusals were collected daily before morning feeding, weighed, and dried in a forced-air oven at 60°C for 72 h to determine DM content [19]. Dry matter intake (DMI) was expressed as kg/day, percentage of LW, and relative to metabolic LW (g/kg LW^0.75^). Nutrient intake values for OM, CP, ADF, and NDF were also calculated to evaluate treatment effects on nutrient consumption.

### Digestibility assessment and analytical procedures

Digestibility was determined by collecting total fecal matter during weeks 5, 9, and 13 of the experiment, following the procedures described by Moran [16]. Feed intake and fecal output data were used to calculate apparent digestibility coefficients. Feces were collected daily from each bull, weighed, homogenized, and a 10% subsample was stored at −20°C. At the end of each 7-day collection period, subsamples were thawed, pooled by animal, and dried at 60°C for 72 h before analysis. Parameters evaluated included DM, OM, CP, ADF, and NDF digestibility. These measurements enabled assessment of nutrient utilization under the different concentrate formulations and followed methods previously used in feed evaluation studies in Bangkalan [22]. Digestible nutrient intake was also expressed relative to metabolic LW to account for differences in animal size.

### Measurement of growth performance and economic indicators

LWG was calculated as:

LWG (Kg/day) = Final LW – Initial LW / Experimental period

Body weight was measured weekly in the morning before feeding, after fasting. Feed conversion ratio (FCR) was calculated as:

FCR = Daily feed intake / Daily weight gain

Economic performance was assessed using IOFC, as described by Ndaru *et al.* [11]. This parameter considered both the economic value of weight gain and the cost of feed consumed. The equation used was:

IOFC (IDR/day) = (LWG, Kg/day × Price of bulls/Kg LW in IDR) – (DMI, Kg × Feed price/Kg DM in IDR)

### Statistical analysis and modeling approach

Data were analyzed using a mixed-model analysis of variance to evaluate the effects of treatment and breed on the measured variables. Treatment and breed were considered fixed effects, whereas block was treated as a random effect. Statistical analyses were performed using the MIXED procedure of SAS version 9.4 (SAS Institute Inc., Cary, NC, USA) [23]. Least squares means were calculated for each variable, and treatment × breed interactions were evaluated when appropriate. Means were separated using Tukey's adjustment when significant differences were detected. Statistical significance was declared at p < 0.05.

The statistical model used was:

Y_ijkl_ = µ + Bi + Tj + (B × T)ij + Blk + Eijkl

where:

1. Y_ijkl_ = observed value;

2. µ = overall mean;

3. Bi = effect of breed;

4. Tj = effect of dietary treatment;

5. (B × T)ij = breed × treatment interaction;

6. Blk = block effect based on initial body weight class;

7. Eijkl = residual error.

The breed × treatment interaction was tested and found to be nonsignificant (p > 0.05) for all major response variables, including intake, digestibility, LWG, FCR, and IOFC. Therefore, interaction effects were omitted from the final model and only the main effects are presented.

## RESULTS

### Nutrient composition of feed ingredients

The chemical composition analyses of each feed ingredient revealed substantial variation in nutrient profiles, reflecting the distinct functional contributions of forage and concentrate components to the overall ration (Table 2).

**Table 2:** Nutrient contents of ingredients used in this study.

**Ingredients**	**DM**	OM*	CP*	EE*	CF*	**NFE**	TDN*	ADF*	NDF*
Native grass	30.27	93.56	6.44	2.21	34.07	45.82	62.6	29.78	68.85
Rice bran	90.41	88.22	7.57	12.79	19.09	48.77	74.1	13.20	10.26
Cassava waste	87.30	87.93	2.14	3.23	0.57	81.99	84.1	21.23	55.88
Cracked corn	89.23	94.91	10.10	5.24	2.50	67.15	83.8	32.45	59.34
Tofu waste	87.12	92.48	18.56	23.89	13.59	36.44	76.3	18.06	38.76
Copra meal	88.17	92.40	25.63	10.74	20.79	44.68	67.3	36.50	54.70
Mineral mix	–	–	–	–	–	–	–	–	–

Values are expressed on a dry matter basis. Total digestible nutrients = 5.307 + 0.2492CF + 0.4119CP + 1.444EE + 0.937NFE [16].

Native grass, which served as the basal forage across all treatments, exhibited a low CP content of only 6.44%. These values indicate limited nutritive value, particularly when compared with concentrate ingredients. The grass also contained high CF (34.07%) and elevated ADF (29.78%) and NDF (68.85%) fractions, indicating substantial structural fiber content that is likely to restrict intake. These characteristics are consistent with previous assessments of forage quality in Bangkalan Regency [19], where high-fiber native grasses commonly limit voluntary consumption and contribute minimally to growth performance.

In contrast, rice bran exhibited relatively favorable nutrient characteristics, and its EE (12.79%) and TDN (74.1%) contents reflect its function as a readily available source of digestible energy. The ingredient's low ADF (13.20%) and very low NDF (10.26%) indicate high digestibility with minimal structural fiber load, thereby enhancing energy supply without substantial ruminal fill constraints. Cassava waste emerged as a highly fermentable energy source, as indicated by its high NFE (81.99%) and TDN value (84.1%). Although the CP content was low (2.14%), the ingredient provided considerable digestible energy potential. Cassava waste contained a moderate ADF (21.23%) and a higher NDF (55.88%), suggesting abundant structural fiber but a sufficiently low proportion of lignified material to support moderate digestibility. The presence of rapidly fermentable carbohydrates, together with moderate fiber levels, necessitated complementary inclusion of protein-rich ingredients or rumen-degradable nitrogen sources.

Tofu waste and copra meal supplied most of the CP within the concentrate mixtures. Tofu waste contained 18.56% CP and a substantial EE fraction of 23.89%, highlighting its dual role in supplying protein and lipid-derived energy. Copra meal provided an even higher CP (25.63%) but was accompanied by elevated CF (20.79%) and ADF (36.50%), thereby moderating its digestibility. Nevertheless, copra meal remained a valuable protein source within appropriate inclusion levels. Cracked corn provided additional digestible energy through its high NFE (67.15%) and TDN (83.8%), although its relatively high NDF content (59.34%) indicated appreciable fiber content.

### Nutrient composition of concentrate mixtures

The varying proportions of cassava waste and tofu waste in concentrate treatments C1–C4 resulted in predictable nutrient shifts (Table 3).

**Table 3:** Nutrient composition of concentrate mixtures

**Chemical composition**	**C1**	**C2**	**C3**	**C4**
DM (%)	87.44	87.45	87.46	83.70
OM*	91.62	91.20	90.78	90.09
CP*	14.10	13.28	12.48	11.33
EE*	16.05	15.02	13.99	11.93
CF*	9.03	8.38	7.73	6.49
NFE*	50.38	52.44	54.50	54.83
TDN*	75.37	75.76	76.16	73.27
ADF*	45.55	46.40	47.25	48.53
NDF*	24.62	24.77	24.93	25.41

Values are expressed on a dry matter basis. Total digestible nutrients = 5.307 + 0.2492CF + 0.4119CP + 1.444EE + 0.937NFE [16].

DM content ranged from 87.46% in C3 to 83.70% in C4, with the reduction in C4 attributable to the higher moisture content of cassava waste. OM content decreased slightly from C1 to C4, reflecting the influence of ingredient moisture and mineral fractions. CP decreased progressively from 14.10% in C1 to 11.33% in C4 as the contribution of tofu waste declined. EE exhibited a similar pattern, decreasing from 16.05% in C1 to 11.93% in C4, consistent with the replacement of high-fat tofu waste by low-fat cassava waste.

CF decreased from 9.03% to 6.49% as tofu waste levels decreased, whereas ADF and NDF exhibited slight increases because of the additional structural carbohydrates supplied by cassava waste. The NFE value increased from 50.38% in C1 to 54.83% in C4, indicating a progressive increase in readily available carbohydrates as cassava inclusion increased. TDN values remained relatively stable (73.27%–76.16%), suggesting that changes in ingredient composition did not compromise dietary energy density. These findings demonstrate that despite variations in nutrient contributions, the four concentrate formulations provided comparable metabolizable energy.

### DMI and nutrient intake

Statistical analysis demonstrated significant effects of treatment and breed on total DMI (p < 0.05; Table 4).

**Table 4:** Dry matter intake and nutrient intake.

**Variables**	**Breeds**	**T1**	**T2**	**T3**	**T4**	**SEM**	**p (T)**	**p (B)**	**p (T × B)**
Total DMI (kg/day)	Madura	4.08	4.63	5.08	5.06	0.243	*	*	ns
	Madrasin	5.91	6.82	6.86	7.19				
Native grass (kg/day)	Madura	0.98	1.12	1.19	1.17	0.663	ns	ns	ns
	Madrasin	1.15	1.25	1.21	1.35				
Concentrate (kg/day)	Madura	3.10	3.51	3.89	3.89	0.191	**	**	ns
	Madrasin	4.76	5.67	5.65	5.84				
Total DMI (% LW)	Madura	1.75	1.92	1.81	1.87	0.070	ns	ns	ns
	Madrasin	1.83	2.01	1.82	2.00				
Native grass (% LW)	Madura	0.33	0.36	0.35	0.36	0.021	ns	ns	ns
	Madrasin	0.35	0.37	0.32	0.38				
Concentrate (% LW)	Madura	1.42	1.56	1.46	1.51	0.054	ns	ns	ns
	Madrasin	1.47	1.64	1.50	1.62				
DMI (g/kg LW0.75)	Madura	72.89	80.45	80.42	79.23	2.947	ns	ns	ns
	Madrasin	77.47	86.41	83.61	87.01				
OM intake (kg/day)	Madura	4.03	4.27	5.03	5.02	0.207	**	**	ns
	Madrasin	5.25	6.14	6.06	6.31				
CP intake (kg/day)	Madura	0.67	0.71	0.68	0.62	0.268	**	**	ns
	Madrasin	0.74	0.82	0.77	0.74				
ADF intake (kg/day)	Madura	1.45	1.63	1.69	1.69	0.006	**	**	ns
	Madrasin	1.62	1.89	1.88	2.01				
NDF intake (kg/day)	Madura	2.58	2.93	3.85	3.09	0.117	**	**	ns
	Madrasin	2.88	3.40	3.42	3.67				

*Significantly different. *Highly significant. ns = Nonsignificant. SEM = Standard error of the mean.

Bulls receiving T2, T3, and T4 exhibited greater DMI than those receiving T1, indicating that diets containing higher proportions of cassava waste supported increased feed intake. For example, Madrasin bulls receiving T4 consumed 7.19 kg/day, representing the highest value among all treatments. This increased consumption may be attributed to improved palatability and enhanced ruminal fermentation, both associated with higher concentrations of soluble carbohydrates.

Breed exerted a consistent effect, with Madrasin bulls consuming significantly more feed than Madura bulls across all treatments. This pattern was observed for total intake (kg/day), percentage of LW, and metabolic LW. For example, Madrasin bulls receiving T2 consumed 86.41 g/kg LW^0.75^ compared with 80.45 g/kg LW^0.75^ in Madura bulls. These differences in feed intake were attributable to the larger body size and higher metabolic requirements of Madrasin bulls.

DMI from native grass was not significantly affected by treatment or breed (p > 0.05), indicating that forage intake remained relatively constant regardless of concentrate composition. This finding highlights the limited nutritional adaptability of native grass under the feeding conditions used. In contrast, concentrate intake differed significantly among treatments and breeds (p < 0.01). Madrasin bulls consistently consumed more concentrate than Madura bulls, particularly in T2–T4, indicating that concentrate composition rather than forage intake was the principal determinant of nutrient intake variation.

### Nutrient intake

Statistical analysis showed that treatment and breed significantly affected OM, CP, ADF, and NDF intake (p < 0.05; Table 4). OM and CP intake increased with increasing DMI, with Madrasin bulls exhibiting greater OM intake across all treatments and the highest value observed in T4 (6.31 kg/day). ADF and NDF intake were significantly affected by treatment and breed (p < 0.01). Madrasin bulls consistently consumed more ADF and NDF, with the highest values recorded under T4. These findings highlight the greater ruminal capacity of Madrasin cattle and their ability to utilize diets with higher concentrations of structural fiber.

### Nutrient digestibility

Digestibility results indicated significant treatment effects for DM and OM digestibility (p < 0.05; Table 5).

**Table 5:** Digestibility coefficients and digestible nutrient intake

**Variables**	**Breeds**	**T1**	**T2**	**T3**	**T4**	**SEM**	**p (T)**	**p (B)**	**p (T × B)**
Digestibility coefficients (%)									
DM digestibility	Madura	77.90	76.97	75.06	77.76	0.602	ns	**	ns
	Madrasin	79.77	77.58	75.52	76.84				
OM digestibility	Madura	78.23	77.26	76.77	78.67	0.847	ns	**	ns
	Madrasin	81.21	78.02	76.80	77.80				
CP digestibility	Madura	76.85	77.37	76.81	78.78	0.803	ns	**	ns
	Madrasin	78.38	77.28	78.18	79.10				
ADF digestibility	Madura	68.81	69.21	68.99	68.91	0.632	*	*	ns
	Madrasin	71.00	69.98	70.93	70.20				
NDF digestibility	Madura	75.34	74.91	75.14	73.38	1.134	ns	ns	ns
	Madrasin	77.38	76.79	76.27	77.23				
Digestible nutrient intake (g/kg LW^0.75^)									
Digestible DMI	Madura	56.78	61.92	62.80	61.61	2.873	*	*	ns
	Madrasin	61.80	67.04	63.14	66.89				
Digestible OMI	Madura	50.78	54.99	52.54	54.55	2.513	*	*	ns
	Madrasin	55.92	59.82	54.57	57.68				
Digestible CPI	Madura	6.99	7.42	6.64	6.40	0.343	**	**	ns
	Madrasin	7.61	7.91	7.06	6.88				
Digestible ADF intake	Madura	13.48	15.24	14.83	15.27	0.824	**	**	ns
	Madrasin	15.09	16.52	15.64	16.58				
Digestible NDF intake	Madura	26.26	29.65	36.80	29.72	0.344	**	**	ns
	Madrasin	29.23	32.60	30.59	33.30				

*Significantly different. *Highly significant. ns = Nonsignificant. SEM = Standard error of the mean.

Bulls receiving T1 exhibited the highest DM and OM digestibility values, probably because of the greater inclusion of tofu waste, which contributed to higher CP and EE concentrations and promoted improved microbial fermentation. Madrasin bulls generally exhibited numerically greater digestibility values than Madura bulls. However, breed effects were significant only for ADF digestibility, whereas DM, OM, CP, and NDF digestibility were not significantly affected by breed.

CP digestibility remained relatively stable across treatments (76.81%–79.10%), indicating adequate protein supply for ruminal microbial activity in all diets. Similarly, ADF and NDF digestibility were not significantly affected by treatment despite slight increases in fiber fractions in C3 and C4.

Breed effects were observed for ADF digestibility, with Madrasin bulls exhibiting significantly higher values (p < 0.05). This finding suggests superior utilization of structural carbohydrates and is consistent with previous studies demonstrating greater ruminal degradation efficiency of fibrous feedstuffs in crossbred cattle [24].

### Digestible nutrient intake

Digestible DMI was significantly affected by treatment and breed (p < 0.05). T2 resulted in the highest digestible DMI (67.04 g/kg LW^0.75^) in Madrasin bulls, followed by T4, T3, and T1. Similar patterns were observed for digestible OM intake.

Digestible CP, ADF, and NDF intake were significantly influenced by treatment and breed (p < 0.01), with consistently higher values observed in Madrasin bulls. These findings highlight the interaction between intake capacity and digestibility coefficients. Although digestibility remained relatively stable across treatments, the greater DMI observed in Madrasin cattle resulted in higher overall digestible nutrient intake.

### LWG and FCR

Statistical analysis showed that LWG was not significantly affected by breed, treatment, or their interaction (p > 0.05; Table 6), although numerically greater gains were observed in Madrasin bulls, particularly those receiving T4.

**Table 6:** Growth performance and economic outcomes.

**Variables**	**Breeds**	**T1**	**T2**	**T3**	**T4**	**SEM**	**p (T)**	**p (B)**	**p (T × B)**
Initial LW (kg)	Madura	278.6	282.0	307.3	294.4	7.781			
	Madrasin	290.5	314.0	345.0	325.5				
Final LW (kg)	Madura	330.7	339.8	366.3	352.9	7.480			
	Madrasin	356.2	375.8	406.0	395.2				
LWG (kg/day)	Madura	0.62	0.69	0.70	0.70	0.029	ns	ns	ns
	Madrasin	0.78	0.75	0.73	0.83				
FCR	Madura	8.64	8.79	9.36	8.70	0.497	ns	ns	ns
	Madrasin	7.58	9.64	9.64	8.64				
IOFC (IDR/day)	Madura	35,745	36,357	37,651	35,581	2.307	*	*	ns
	Madrasin	42,156	44,456	41,346	40,376				

*Significantly different. *Highly significant. ns = Nonsignificant. SEM = Standard error of the mean.

All four diets supported comparable growth, indicating that variations in the proportions of cassava waste and tofu waste did not compromise nutrient adequacy. This observation is consistent with previous findings showing that appropriately balanced cassava-based diets can support efficient growth [18]. Madrasin bulls tended to exhibit higher average daily gain (0.77 kg/day) than Madura bulls (0.68 kg/day), reflecting their superior growth potential, enhanced intake, and greater nutrient utilization efficiency.

Similarly, FCR was not significantly affected by treatment or breed (p > 0.05), indicating that feed efficiency remained stable among treatments. Both Madrasin and Madura bulls converted feed into body weight with comparable efficiency despite differences in absolute weight gain.

## IOFC

IOFC values were significantly affected by treatment and breed (p < 0.05), whereas the treatment × breed interaction was not significant (p > 0.05). Among treatments, T2 produced the highest average IOFC, followed closely by T3, whereas T4 exhibited slightly lower economic efficiency despite relatively high LWG.

Madrasin bulls achieved a higher average IOFC (IDR 42,083/day) than Madura bulls (IDR 36,333/day). The absence of significant breed × treatment interactions for growth and economic variables indicated that both breeds responded similarly to the dietary treatments. Therefore, treatment effects could be interpreted independently of breed effects under the conditions of the present study. These findings confirm the economic advantage of Madrasin cattle in fattening systems, although the implications for the conservation of indigenous Madura cattle remain important from the perspective of genetic resource preservation [25].

## DISCUSSION

This study characterized the nutritional composition of locally available feed ingredients, evaluated four concentrate formulations varying in the proportions of cassava and tofu waste, and compared the production responses of Madura and Madrasin bulls under smallholder conditions in Bangkalan Regency. The present work provides several original contributions. First, it is among the few *in vivo* studies directly comparing pure Madura and Madrasin bulls that receive identical rations based on local agricultural by-products, thereby demonstrating that breed differences in feed intake and fiber utilization persist even when diets are optimized with cassava and tofu waste. Second, the systematic replacement of tofu waste with cassava waste produced predictable nutrient shifts while maintaining comparable growth performance and IOFC, offering smallholders greater flexibility in ration formulation than previously documented. Third, by conducting the experiment in adapted communal village cattle stalls rather than under research station conditions, the findings possess greater external validity for East Java smallholder systems, an aspect often overlooked in tropical feeding studies. Overall, the results demonstrate that agricultural and agro-industrial by-products, particularly cassava waste, tofu waste, and copra meal, when incorporated into balanced concentrate mixtures, can effectively support cattle production.

### Nutritional characteristics of local feed resources

The nutrient composition of the feed ingredients used in this study illustrates the complementary functional roles required for formulating balanced rations in smallholder production systems. Native grass, which served as the basal forage, had low DM and CP concentrations and high ADF and NDF fractions. These characteristics are consistent with previous reports from Bangkalan Regency [19], where native grasses are abundant but frequently deficient in nutrients necessary to sustain optimum cattle performance. The high structural fiber content of native grass may reduce digestibility and limit voluntary feed intake, thereby confirming that forage alone is insufficient to support maximum growth in either indigenous or crossbred cattle.

In contrast, concentrate ingredients provided complementary nutrient fractions. Rice bran and cracked corn supplied readily digestible energy with relatively low fiber concentrations that favor ruminal degradation. Cassava waste, characterized by high NFE and TDN contents, provided rapidly fermentable carbohydrates that stimulated microbial activity when accompanied by an adequate protein supply. Because of its low CP content, cassava waste required supplementation with protein-rich ingredients such as tofu waste and copra meal. Tofu waste supplied both protein and lipid-derived energy and therefore played an important role in formulations with greater CP concentrations (C1 and C2). Although copra meal was rich in CP, its elevated structural fiber content required careful dietary balancing to prevent reductions in digestibility. Collectively, these ingredients provided complementary energy and protein fractions, supporting previous recommendations regarding the use of cassava-based diets [26, 27].

Across treatments, increasing cassava waste and decreasing tofu waste produced predictable changes in nutrient composition. CP and EE decreased progressively, whereas NFE increased and ADF and NDF increased slightly. Nevertheless, TDN remained within a relatively narrow range (73.27%–76.16%), indicating that overall dietary energy density was maintained. This nutritional consistency provided a suitable basis for evaluating physiological and productive responses to changing ingredient proportions.

### Effects of dietary treatments and breed on feed intake

The concentrate formulation significantly affected DMI and the digestibility of DM and OM. Greater DMI observed in T2–T4 suggests that increasing cassava waste inclusion improved ration palatability and fermentability. Cassava waste contains rapidly fermentable starch and soluble carbohydrates that stimulate microbial proliferation and accelerate ruminal turnover, thereby increasing feed intake. These findings are consistent with previous reports demonstrating that cassava-based supplementation enhances feed intake and ruminal function in tropical cattle systems [24, 28].

Breed differences represented a major source of variation in feed intake. Madrasin bulls consistently consumed more feed across all treatments, reflecting their larger body size and greater nutrient requirements. Previous studies have shown that crossbred cattle with European genetic influence exhibit greater feed intake capacity and enhanced ruminal fermentation efficiency [19, 29]. The greater feed intake observed in Madrasin bulls was primarily attributable to increased concentrate consumption, particularly under T2–T4, suggesting that this breed may be better adapted to diets rich in fermentable carbohydrates. However, when intake was expressed relative to LW or metabolic LW, the higher intake observed in Madrasin bulls was not statistically significant, indicating that body size differences accounted for much of the variation.

Native grass intake did not differ significantly among treatments or breeds, indicating that forage consumption was constrained by the forage's intrinsic structural characteristics rather than by dietary formulation or breed. This finding emphasizes the limited contribution of low-quality forage to productive performance in smallholder systems and highlights the importance of concentrate supplementation for satisfying nutrient requirements.

Greater intake of CP, ADF, and NDF by Madrasin bulls, together with their improved utilization of fiber fractions, reflects their superior digestive capacity and greater ability to tolerate diets containing higher structural fiber concentrations. These physiological advantages likely contribute to the superior productive performance and economic returns observed in this breed.

### Nutrient digestibility and digestible nutrient intake

Digestibility of DM and OM was influenced by dietary treatment, with T1 producing the highest values. This response is likely attributable to the greater CP and EE concentrations in tofu waste-rich formulations, which support microbial proliferation and carbohydrate fermentation. The importance of dietary protein in optimizing microbial activity has been extensively documented [30], and the relatively lower CP concentrations in T3 and T4 may explain the slightly lower digestibility values observed.

Despite these differences, CP, ADF, and NDF digestibility remained relatively stable among treatments. This stability indicates that moderate variations in protein and fiber concentrations did not exceed thresholds that could impair microbial efficiency. In addition, the relatively constant TDN values among diets likely maintained adequate microbial energy supply, thereby sustaining nutrient digestibility.

Breed effects on digestibility were selective. Overall, DM, OM, CP, and ADF digestibility did not differ significantly between breeds; however, NDF digestibility was significantly greater in Madrasin bulls. Although microbial populations were not evaluated in the present study, this observation supports previous reports indicating that crossbred cattle possess greater ruminal capacity and enhanced microbial activity, thereby facilitating more efficient degradation of structural carbohydrates [30]. Improved fiber digestibility constitutes an important physiological advantage in production systems where moderate- to low-quality forage predominates.

Patterns of digestible nutrient intake reflected the combined influence of feed intake and digestibility. Although digestibility values were numerically highest in T1, digestible nutrient intake was greater in T2 and T4 because of higher feed consumption. These findings highlight the importance of considering both nutrient digestibility and animal feeding behavior when evaluating nutrient availability and productive performance.

### Growth performance and feed efficiency

Differences in nutrient intake did not translate into significant differences in growth performance among treatments. All four concentrate formulations supported similar LWG, indicating that variations in the proportions of cassava and tofu waste did not compromise nutrient adequacy. These results are consistent with previous studies demonstrating that properly balanced cassava-based diets can sustain satisfactory growth in tropical cattle systems [24]. The similarity in TDN values among treatments likely contributed to the absence of treatment effects on growth performance.

Although treatment effects were not significant, breed influenced growth performance. Madrasin bulls achieved a higher average daily gain (0.77 kg/day) than Madura bulls (0.68 kg/day), reflecting their superior genetic potential for growth and nutrient utilization. Similar observations have been reported for indigenous and European crossbred cattle in Indonesia [29].

FCR did not differ significantly among treatments or breeds, indicating that cattle converted feed into body weight with comparable efficiency despite differences in nutrient intake and growth. Therefore, the superior performance of Madrasin bulls was primarily attributable to greater nutrient intake and improved fiber utilization rather than to enhanced feed conversion efficiency.

### Economic implications and breed conservation

Improved productive performance has limited practical significance unless accompanied by favorable economic returns. Therefore, economic performance was evaluated using IOFC. The results demonstrated that all dietary treatments were profitable and that no concentrate formulation conferred a marked economic advantage over the others. Despite differences in ingredient composition, feed costs and growth responses remained sufficiently balanced to produce similar financial returns. This finding is particularly important for smallholder farmers who operate under fluctuating feed prices and seasonal variation in the availability of agricultural by-products.

Breed exerted a significant effect on economic performance, with Madrasin bulls generating greater IOFC values than Madura bulls owing to their superior growth performance and higher market value. These findings are consistent with previous studies conducted in East Java showing that well-balanced diets combined with high-growth genotypes enhance profitability [24, 31].

The consistently favorable performance observed across treatments demonstrates the robustness of cassava-protein meal combinations in supporting growth and confirms previous findings regarding the effectiveness of cassava-based rations [18, 24]. Furthermore, the economic neutrality of the four formulations provides flexibility in feed resource utilization, enabling farmers to adjust rations according to ingredient availability without sacrificing profitability. Such flexibility represents an important advantage for smallholder production systems characterized by limited purchasing power and inconsistent feed supply.

The breed differences observed in the present study reveal a persistent productivity gap between Madura and Madrasin cattle under improved nutritional management. The advantages of crossbred cattle in terms of LWG and IOFC clearly provide strong incentives for crossbreeding in regions where Madura cattle predominate. Nevertheless, while crossbred animals offer short-term gains in productivity, long-term sustainability must also consider the conservation of the unique adaptive characteristics of Madura cattle. Economic benefits should therefore be evaluated within the broader context of national genetic resource conservation. Excessive dependence on crossbred animals could accelerate the decline in genetic purity of Madura cattle, a concern that has been highlighted previously [25].

### Practical implications, limitations, and future perspectives

Collectively, the results demonstrate the considerable potential of locally available agricultural by-products to support efficient and economically viable cattle production in Bangkalan Regency. Greater utilization of these resources can strengthen feed self-sufficiency and reduce dependence on commercial feed ingredients [27, 32]. Concentrate formulations based on cassava waste, tofu waste, and copra meal provided adequate nutritional support for both indigenous and crossbred cattle, emphasizing that local feed resources remain underutilized but highly promising.

Nevertheless, several limitations should be acknowledged. The experiment involved a relatively small number of animals per treatment combination and was conducted over a relatively short period at a single location. Consequently, caution should be exercised when extrapolating the findings to broader production environments. Future studies should involve larger populations and evaluate the effects of seasonal variation in feed prices and availability. Additional investigations should also incorporate carcass characteristics and methane emissions to provide a more comprehensive assessment of production efficiency and environmental sustainability.

Overall, the integrated evaluation of feed resources, nutrient composition, intake behavior, digestibility, growth performance, and economic returns provides valuable evidence for designing feeding strategies that balance productive efficiency with the conservation of local genetic resources. Sustainable cattle production in Bangkalan Regency will depend on feeding innovations that maximize the use of local agricultural by-products while simultaneously maintaining breeding programs that preserve the genetic integrity of Madura cattle.

## CONCLUSION

This study demonstrated that locally available agricultural and agro-industrial by-products can be effectively utilized to formulate nutritionally balanced and economically viable concentrate rations for cattle raised under smallholder conditions in Bangkalan Regency, Indonesia. Progressive replacement of tofu waste with cassava waste produced predictable changes in nutrient composition, characterized by reduced CP and EE contents and increased NFE and fiber fractions, while maintaining relatively stable dietary energy density. Consequently, all four concentrate formulations supported comparable digestibility, LWG, FCR, and IOFC, indicating that varying proportions of cassava waste and tofu waste can be used without compromising productive performance.

Breed exerted a more pronounced influence than dietary treatment on nutrient intake and productive responses. Madrasin bulls consistently exhibited greater DMI, higher digestible nutrient intake, superior fiber utilization, and greater LWG and IOFC than Madura bulls. These findings confirm the superior growth potential and economic advantages of crossbred cattle under improved feeding systems. Nevertheless, the ability of indigenous Madura cattle to maintain satisfactory performance under the same nutritional conditions highlights their continued importance for sustainable cattle production in resource-limited, harsh tropical environments.

A major strength of this study lies in its integrated evaluation of feed composition, intake behavior, digestibility, growth performance, and economic returns under realistic communal village production conditions rather than under research station environments. This approach enhances the applicability of the findings to practical smallholder systems. In addition, the direct comparison between pure Madura and Madrasin bulls that receive identical locally formulated diets provides novel information on breed-specific responses to agricultural by-product-based feeding strategies.

In conclusion, concentrate formulations incorporating cassava waste, tofu waste, and copra meal can effectively support productive and profitable cattle production under smallholder conditions. While Madrasin bulls exhibited superior productive and economic performance, the conservation of indigenous Madura cattle remains essential because of their unique adaptive characteristics and genetic value. Sustainable cattle development in Indonesia will therefore require feeding innovations that maximize the use of local by-products while simultaneously preserving valuable indigenous cattle genetic resources.

## DATA AVAILABILITY

The supplementary data can be available from the corresponding author.

## GENERATIVE AI DECLARATION

The authors declare that generative artificial intelligence (AI) tools were used solely to improve language, grammar, and readability during manuscript preparation. All scientific content, data analysis, interpretation of results, and conclusions were developed and verified by the authors. The authors take full responsibility for the accuracy, integrity, and originality of the work presented, and no AI tool was listed as an author.

## AUTHORS’ CONTRIBUTIONS

KK, MM, and PHN: Conceptualized and designed the study. KK and MM: Supervised the feeding trial and interpreted the data. PHN: Conducted the literature review, organized the manuscript structure, drafted the original manuscript, and managed the references. ADK: Assisted with data collection, organization of feed and nutrient composition data, preparation of statistical data, and manuscript formatting. STM and KH: Contributed to the interpretation of the findings, critically reviewed and edited the manuscript, and provided important intellectual input. All authors have read and approved the final version of the manuscript and agree to be accountable for all aspects of the work.
